# MimoSA: a system for minimotif annotation

**DOI:** 10.1186/1471-2105-11-328

**Published:** 2010-06-16

**Authors:** Jay Vyas, Ronald J Nowling, Thomas Meusburger, David Sargeant, Krishna Kadaveru, Michael R Gryk, Vamsi Kundeti, Sanguthevar Rajasekaran, Martin R Schiller

**Affiliations:** 1 Department of Molecular, Microbial, and Structural Biology, University of Connecticut Health Center, 263 Farmington Ave. Farmington, CT 06030-3305 USA; 2 School of Life Sciences, University of Nevada Las Vegas, 4505 Maryland Pkwy. Las Vegas, NV 89154-4004 USA; 3 Department of Computer Science and Engineering, University of Connecticut, 371 Fairfield Rd., Storrs, CT 06269-2155 USA

## Abstract

**Background:**

Minimotifs are short peptide sequences within one protein, which are recognized by other proteins or molecules. While there are now several minimotif databases, they are incomplete. There are reports of many minimotifs in the primary literature, which have yet to be annotated, while entirely novel minimotifs continue to be published on a weekly basis. Our recently proposed function and sequence syntax for minimotifs enables us to build a general tool that will facilitate structured annotation and management of minimotif data from the biomedical literature.

**Results:**

We have built the MimoSA application for minimotif annotation. The application supports management of the Minimotif Miner database, literature tracking, and annotation of new minimotifs. MimoSA enables the visualization, organization, selection and editing functions of minimotifs and their attributes in the MnM database. For the literature components, Mimosa provides paper status tracking and scoring of papers for annotation through a freely available machine learning approach, which is based on word correlation. The paper scoring algorithm is also available as a separate program, TextMine. Form-driven annotation of minimotif attributes enables entry of new minimotifs into the MnM database. Several supporting features increase the efficiency of annotation. The layered architecture of MimoSA allows for extensibility by separating the functions of paper scoring, minimotif visualization, and database management. MimoSA is readily adaptable to other annotation efforts that manually curate literature into a MySQL database.

**Conclusions:**

MimoSA is an extensible application that facilitates minimotif annotation and integrates with the Minimotif Miner database. We have built MimoSA as an application that integrates dynamic abstract scoring with a high performance relational model of minimotif syntax. MimoSA's TextMine, an efficient paper-scoring algorithm, can be used to dynamically rank papers with respect to context.

## Background

Minimotifs are short peptide sequences that are the recognition elements for many protein functions. These short sequences are responsible for protein interaction interfaces involving other proteins (or molecules) in cells, trafficking proteins to specific cellular compartments, or serving as the basis for enzymes to post-translationally modify the minimotif sequence. At present, many minimotif instances and consensus sequences are collected into a wide spanning set of relatively small databases such as MnM, ELM, Domino, PepCyber, and ScanSite [1-5]. Most databases focus on specific subsets of minimotifs. For example, Phospho-ELM has merged with PhosphoBase as a database that focuses on instances of phosphorylation on proteins [6]. Likewise, ScanSite largely concentrates on protein interaction minimotifs for a small subset of domains. In addition to these databases, recent years have seen increased publication rates of high throughput studies that generate minimotif data. Despite this growth in information, many of the reported minimotif attributes have yet to be integrated into any database.

The goal of the MnM project is to integrate well-structured data for a set of defined attributes of minimotifs in a single, non-redundant data repository with high accuracy. The number of reports of minimotifs in the literature has continued to grow since the late 1980's, recently with more rapid growth due to high throughput functional peptide screens. Previously, we showed that the several thousand minimotifs in MnM can be discretized into a structured syntax which can be directly enforced and modeled in a relational database [1,7]. Through this process, we recognized the need for a system that manages minimotif annotation, which would help identify papers, reduce the time required for manual annotation, reduce errors, duplications and ambiguities, and aids in maintenance of the database.

Currently, there are no bioinformatics tools designed for annotating minimotifs from the literature. Most reported annotation methodologies concentrate mainly on genomes and proteome scale data [8-10]. A proposed stratification of annotation efforts refers to sequence-based annotation as the first dimension of genome annotation which defines components [11]. The second dimension can be considered those annotations that focus on component interactions. This is exemplified by the human kinome and other types of functional annotations in the SwissProt and Entrez Gene databases [12,13]. Annotation of minimotifs can be considered a second dimension annotation.

In considering whether to design a novel minimotif annotation system or adapt an existing annotation system used for another purpose, we identified a number of requirements to facilitate accurate, non-redundant, and efficient annotation of minimotif literature. We wanted the system to interface with a relational database that enforces controlled vocabularies from external databases and eliminates duplication. The system should be able to read, write, and edit entries in a database. The system should display papers that have been and are yet to be annotated, as well as support database-driven machine learning that scores papers for minimotif content, paper sorting, and paper filtering. The system should also have the capability to track annotations from multiple annotators. Finally, the system should be capable of accepting the fine-grained information content of minimotifs, in a structured and comprehensive manner.

Despite advances in management and mining of scientific literature, no tool existed that met the requirements we required for accurately annotating minimotif data. For example, each of the existing annotation tools such as MIMAS, Textpresso and Biorat only addresses a subset of the above requirements [14-16].

In this paper, we describe MimoSA, a **Mi**ni**mo**tif **S**ystem for **A**nnotation designed for managing and facilitating minimotif annotation. MimoSA allows for minimotif-centric analysis of PubMed abstracts and annotation of minimotifs. MimoSA's contents are entirely database driven, thus enabling its adaption as an annotation tool for other information spaces that require extraction of information from the primary literature.

## Implementation

We present the generalizable architecture and implementation of MimoSA, an application, which allows minimotif annotations to be entered, reviewed, edited, approved by multiple users, and disseminated through the publically-available MnM web application. We also describe a generalizable paper-scoring algorithm and its implementation for ranking papers that contain minimotifs. By embedding this methodology into MimoSA, PubMed abstracts can be scored and associated papers can be ranked based on the presence of minimotif information content.

MimoSA was developed in Java http://java.sun.com and interfaced with a MySQL database http://www.mysql.com using the Hibernate object-relational mapper http://www.hibernate.org. MimoSA was built to interface with the MnM relational database, which has been expanded to include the ability to store information about papers to be annotated and the relationships between minimotif annotations and their source papers [7]. The graphical user interface (GUI) was developed using Swing http://java.sun.com/docs/books/tutorial/uiswing. Supporting applications used for offline data processing were also developed in Java. These applications identify new keywords and terms used to highlight text in the abstract display window and download content and metadata from PubMed for papers added into the system. For these features, we have relied extensively on the PubMed Application Programming Interface (API) and Remote Procedure Call (RPC) library.

Unlike other annotation and text mining systems, the data artifacts produced by MimoSA are accessible by an API, which is syntax-driven and strongly typed. This allows for high-precision annotation of articles that is not coupled to any one data repository. Thus, MimoSA may easily be configured, for example, to save annotations to an XML document or text file by simply modifying the data access layer implementation.

The generality of the MimoSA application enables its adaptation to other databases and other knowledge domains. This was a consideration made during the development of MimoSA, so as to more broadly enable adaption to other bioinformatics projects.

## Results

### MimoSA prototype design

The primary function of MimoSA is to support the process of annotating functional minimotifs and their metadata from the primary literature. Secondary functions include minimizing user errors and data redundancy, improving annotation efficiency through techniques such as automated motif/activity/target suggestions, and aiding in the identification of papers containing minimotif content through a machine learning-based ranking system. MimoSA features distinct components and algorithms, which streamline these processes.

**Figure 1:**
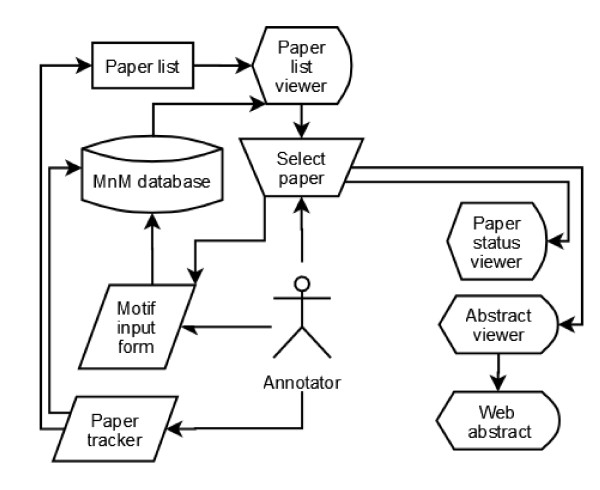
**General architecture of MimoSA**.

The general annotation workflow is as follows (see Fig. 1): Using the MimoSA client software, the annotator accesses the server housing the MnM database. The user selects a paper for annotation using the Paper List Viewer. Selection of a paper automatically triggers the opening of the Abstract Viewer and the Minimotif Annotation Form and directs an external web browser to online versions of the abstract and full text paper, if available. Based on the information in the viewers, the Minimotif Annotation Form is used to modify an existing or enter a new minimotif annotation, which is then committed to the database. The annotation status of the paper is updated using the Paper Tracker Form.

The components of MimoSA can be broken up into three functional categories: MnM database management tools, minimotif annotation tools, and paper management tools. Descriptions of each component follow.

**Figure 2:**
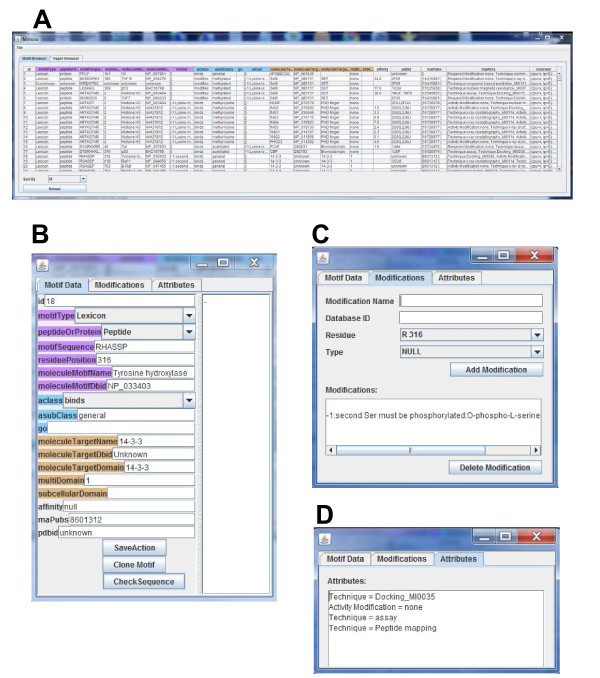
**Screen shots of MimoSA application database management windows**. **A**. Motif Browser shows attributes of all minimotifs in the MnM database. **B**. Minimotif Data editing or entry form for entering information in the MnM database. **C**. Modification form for entering minimotif modification or activity modification attributes into MnM **D**. Attribute for adding or editing supporting experimental techniques, structure accession numbers, and other auxiliary attributes including comments about the annotation.

The database management tools consist of a minimotif browser and a minimotif editor. The minimotif browser shown in Fig. 2A displays all minimotif annotations in the MnM database and associated attributes in a scrollable window that also displays the total number of minimotifs. A Paper Browser is accessed from a tab and gives a list of papers that need annotation. From the paper or minimotif browsers, a Minimotif Annotation Form can be launched by double clicking a row to enter a new or modify an existing minimotif annotation (Fig. 2B-2D). This opens a tabbed frame where all the minimotif attributes are displayed and can be added or changed. Minimotif annotations can be selected for exportation as Comma-Separated Value (CSV) files for external manipulation. Likewise, an import function allows import from a CSV file. The minimotif annotations in the browser can be sorted based on a number of different attributes from a drop-down menu.

The minimotif annotation tools consist of the Minimotif Annotation Form, the Abstract Viewer, and the Protein Sequence Validator. Multiple forms can be displayed at once. On the Minimotif Annotation Form, there is a "clone" function, which opens a new instance of the form pre-filled with all of the minimotif-syntactical attributes except the minimotif's sequence and position. This is intended to facilitate more efficient annotation of high-throughput papers for minimotif discovery (e.g. phage display), where several attributes of a minimotif are varied in a controlled fashion, thus generating a broad landscape of similar minimotifs with subtle variations [17,18].

**Figure 3:**
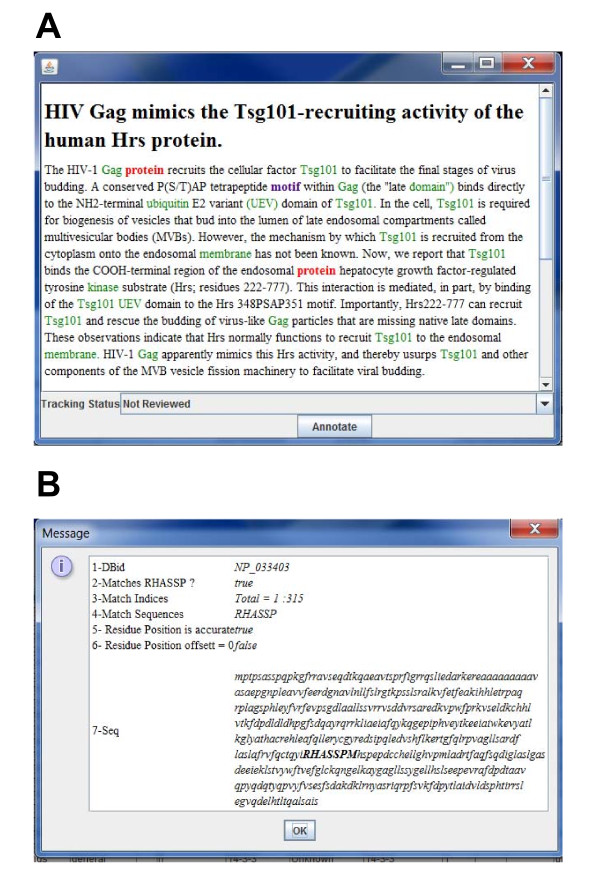
**Screenshot of MimoSA abstract and protein sequence viewers**. **A**. Abstract Viewer shows the abstract of the paper selected. Words that match existing minimotif attributes are color-coded. **B**. Protein sequence viewer window shows the sequence of the protein having the accession number entered in the Minimotif Data entry form. Any minimotif entered in this form is highlighted in the sequence.

To assist the annotator in filling out the form, multiple types of support are provided. Double-clicking on any entry field in the form will display a context menu that gives the suggested choices based on relevant content in the MnM database. In the Modification tab, selecting a modification from the context menu will populate a different field in the form with a PSI-MOD accession number. The Abstract Viewer (Fig. 3A) automatically displays the PubMed abstract of paper that has been selected and highlights keywords and terms in different colors based on attribute entries in the database. The coloring scheme is minimotif (purple), activity (blue), target (orange), putative minimotif (red), affinity (yellow), protein domain (green); if the word "motif" is present, it is bolded. Selection of a paper with a right click also opens the abstract on the PubMed web site and a full text version of the paper, if available, in a web browser. This enables efficient access to full text papers and to other NCBI data using the "Links" hyperlink. Linked data of interest to the annotator includes structure and RefSeq accession numbers.

Another component that assists annotators is the Protein Sequence Validation function (Fig. 3B). Once an accession number has been entered, the protein sequence is automatically retrieved from a local version of public databases such as NCBI and displayed in the Protein Sequence Window. Once loaded, the position of the minimotif in the protein sequence is bolded. This ensures that the minimotif is indeed present in the selected protein.

**Figure 4:**
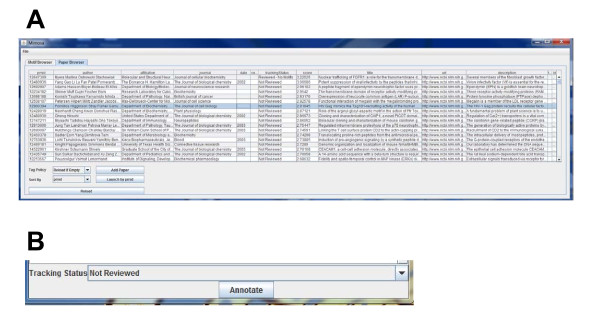
**Screenshot of MimoSA paper browser and paper tracking windows**. **A**. Paper Browser Window allows display of attributes for all papers in the MnM database. **B**. Paper Review Status Window shows review event history of papers in the database.

The paper management tools consist of the Paper Browser, Paper Status Window, and Paper Ranking components, which are addressed later. The Paper Browser shown in Fig. 4A can be used to manage millions of papers. The Paper Browser displays metadata about the PubMed abstracts of all papers entered into a table of the MnM database. The metadata includes PubMed ID, authors, affiliation, journal, publication year, comments, tracking status, paper score, title, URL, abstract, and database source. A paper score (discussed later) is used as a default sort parameter, although the entire table can also be sorted by PubMed ID, paper status, PubMed identifier, publication year, or journal using a pulldown menu. Since the table containing papers has more than 120,000 tuples, only the first 1,000 results of any sort are shown. When a PubMed identifier is entered and the "Add Paper" button is selected, the associated paper is retrieved from NCBI and inserted into the database. Any abstract can be retrieved for review by selecting the "Launch by PubMed ID.".

The Paper Status Window, a subcomponent of the Abstract Window, is used to track the annotation status of papers (Fig. 4B). Each time a paper is reviewed and the user updates the status of the paper, a "review event" is created and appended to the paper's history, which is stored in the database; the review event identifies the annotator and current status of the paper. Papers can be assigned one of a number of statuses shown in Table 1 that correlate with different tracking functions.

**Table 1:** Paper tracking status definitions

Name	Description
not reviewed	For papers that have not yet been received.

Reviewed no minimotifs	For papers that were reviewed and do not contain minimotifs.

reviewed for some minimotifs	For papers that were reviewed, but for which not all of the minimotifs from the paper have been annotated.

reviewed for all minimotifs	For papers that were reviewed and and for which all minimotifs have been annotated.

group review	For papers with questionable interpretation that require discussion by the annotation group.

no electronic version	For papers for which an electronic version is not available.

minimotif present but not annotated	For papers that have a minimotif, but have not yet been annotated.

### Modification of the minimotif miner data model and syntax

In order to better exploit MimoSA's functionality and facilitate unambiguous and accurate annotation, we recognized that some changes to the model we previously presented were required [7]. Our minimotif syntax defined the *motif source *as the protein that contains the minimotif. However, a consensus minimotif definition such as [RK]xx[RK] can have multiple occurrences in a *minimotif source *so we needed to specify a position for the first minimotif residue relative to the protein sequence start position in the corresponding sequence file specified by a protein sequence accession number. Another change we considered is that experiments, which contribute to minimotif definitions may either use peptides or full length proteins. We think it is important to specify this as an attribute since the two sources represent very different chemical entities. Finally, we have started using PSI-MOD and GO controlled vocabularies for indicating activities and post-translational modifications of minimotifs.

### Identification of papers with minimotif content

The MnM database contains many papers that were previously annotated for minimotif content, but many more papers have yet to be annotated. PubMed contains well over 19 million abstracts of scientific papers. Only those papers that have minimotif content are useful for annotation. Our first approach to pare down the paper list used keyword searches to identify papers, which were likely to contain minimotif content; however, this approach was not efficient. Therefore, we developed new strategies and an efficiency metric for the evaluation and comparison of these strategies (see Additional File 1).

We initially evaluated six general strategies: Keywords/Medical Subject Headings (MeSH), date restriction, forward and reverse citations, authors with affiliations, and minimotif regular expressions. A detailed description of the strategies and results are presented in Additional File 1. These strategies were evaluated using a Minimotif Identification Efficiency (MIE) score, which is defined as the percentage of papers that contain minimotifs. Collectively, these strategies provided a list of approximately 120,000 abstracts, of which ~30% were expected to contain minimotifs based on extrapolation.

### Design and training of the TextMine algorithm that scores papers for minimotif content

We wanted to score and rank these papers as a means to better identify the ~30% that contain minimotifs and develop a strategy for scoring all PubMed papers that can be used for future maintenance of the MnM database. To rank papers for minimotif content, we designed the Paper Scoring (PS) algorithm and trained the algorithm using structured data for defined paper sets in the MnM database.

The basic problem of interest can be stated as follows: given a research article (or an abstract), automatically rank the article by its likelihood of containing a minimotif. We used a subset of papers as a training set for training the PS algorithm. Each article in a research article collection *A*, which is used for training, is read by hand and given a score of either 0, indicating that the paper does not contain minimotifs, or 1, indicating that the paper has at least one minimotif. A similar algorithm has been employed to characterize unknown microorganisms [19]. A crucial difference between the PS algorithm and that of Goh, *et al*., is that the PS algorithm provides an ordering of the papers instead of using a filter threshold.

The workflow for this phase consists of the following steps: We start with disjoint sets *P*, *N*, and *T *of abstracts, which are positive, negative, or not reviewed for minimotif content, respectively. Let *W *be the ordered term vector found by taking all significant words (e.g. words like "the", "of", "new" etc., that have no discriminatory value between *P *and *N*) from the documents of sets *P and N*. For each word *w *in *W *and each article *a *in *P *we divide the number of instances of *w *by the size of *a*: this is the enrichment of *w *in *a*. Then, we sum these enrichments over all *P *and divide by the size of *P *to obtain an overall enrichment of *w*. We repeat this over set *N*, and subtract the result from *wp *to arrive upon a "score" for word *w*, which ranges from -1 to 1. Higher values indicate more positive association with minimotif content. We now have a vector of decimal "scores", which has the same dimension as *W*, with one entry per term in the term vector. Call this vector *S*.

Now, we compute a score for each unknown paper by combining word scores. This phase consists of the following steps.

1) Scan through the paper (or abstract) to count how many times each word *w *of *W *occurs in this article.

2) Construct a vector *v *of all values from (1) in which the order corresponds with *S*.

3) Compute the correlation between *v *and *S *and obtain a Pearson's correlation coefficient *pc *for each paper. If *X *and *Y *are any two random variables, then the Pearson's correlation coefficient between *X *and *Y *is computed as  where *μ*_*X *_is the expected value of *X*, *μ*_*Y *_is the expected value of *Y*, *σ*_*X *_is the standard deviation of *X*, and *σ*_*Y *_is the standard deviation of *Y*.

4) Thus, we have now computed a "score" of the article, which is the Pearson's correlation coefficient between the scored words from the training set *W *and respective enrichments of those words in the article *n*.

The Paper Scoring (PS) algorithm's pseudo code is provided in the Additional File 1. The correlation coefficients for the lexemes range from -1.000 to 1.000. This score positively correlates with the presence of minimotif content, as expected.

### Paper ranking and evaluation of the paper scoring algorithm

The algorithm above is packaged as an independent application, TextMine, which can be used in conjunction with MimoSA (or as a standalone open source java application which can be integrated with any annotation or analysis pipeline). For the test set, we selected 91 new articles, which we determined to either have or not have minimotif content and were disjoint from the training sets. The basis for all testing of the TextMine application was derived from correlations of TextMine scores to this set.

The TextMine website and package provides a test data set which reproduces our analysis for a set of test papers. The current version of MimoSA, utilized for MnM annotation, uses scores from TextMine calculated for 120,000 abstracts for paper sorting.

### Paper scoring algorithm and training set size

Since the purpose of the algorithm is not simply to rank papers, but rather, to rank papers with increasing sensitivity over time, we evaluated the increase in the algorithms efficacy with respect to larger training sets. We found that there was a degree of variation depending on training set sizes, but that overall, both positive and negative training elements improved the performance (Table 2).

**Table 2:** Larger training set sizes (negative, positive) modestly improve algorithm performance

Negative Papers	Positive Papers	Paper Score
10	100	0.60

20	100	0.63

30	100	0.63

40	100	0.64

10	200	0.56

20	200	0.59

30	200	0.58

40	200	0.60

10	300	0.60

20	300	0.63

30	300	0.64

40	300	0.66

10	400	0.61

20	400	0.65

30	400	0.66

40	400	0.66

For use in testing TextMine's performance relative to the size of the training set the application package includes an iteration module, which allows for specification of the sizes of positive and negative training sets (this iteration package generated the data in Table 2). We recorded the performance for incrementally increased training set sizes, and noted that as the number of either positive or negative training documents increased, a modest performance improvement was observed. The performance of the algorithm is determined by the correlation coefficient between the calculated scores, between -1 and 1, and an actual score, between 0 and 1.

The table indicates that large increases in the number of positive training articles were comparable to small increases in the number of negative training articles, ultimately showing that both had modest increases in value with set size. A positive correlation coefficient between positive or negative training size and the algorithm performance was observed (0.52 and 0.46, respectively). The correlation score between TextMine scores and the training set scores showed modest increases with size (ranging from 0.59 to 0.66 when using 40 negative and 400 positive abstracts).

**Figure 5:**
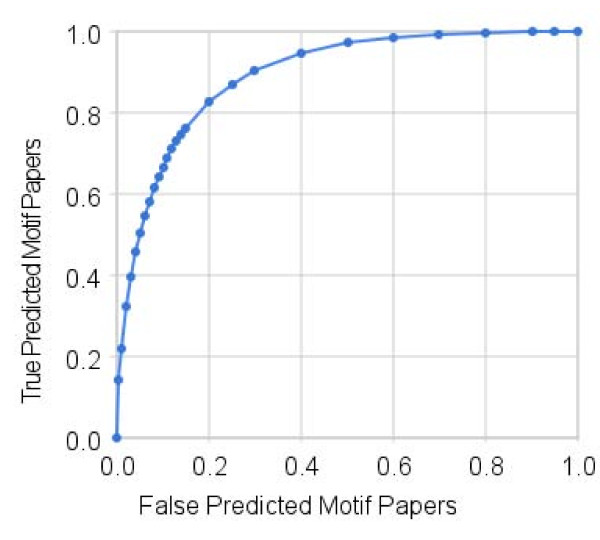
**ROC curve analysis of TextMine results**. ROC curve as a measurement of the sensitivity and specificity of TextMine for a disjoint test set of 91 pre-scored papers. Area under curve = 0.89.

The Receiver Operator Characteristic (ROC) curve is a standard metric for visualizing the sensitivity and specificity of an algorithm, which differentiates two populations. We have also included a ROC curve for the highest scoring training set, which had 400 positive and 40 negative articles. We found that this proportion was not required, and that significant correlations could also be obtained with smaller data sizes, as previously described. This curve is shown in Fig. 5. Notably, the area under the curve was above 0.89, indicating a high correlation between the score magnitude and the presence (1) or absence (0) of a minimotif. This data can be generated using the TextMine package. The steps for reproducing this data are described in the TextMine application package.

Because the general utility of this algorithm far exceeds the field of minimotif annotation, we have released TextMine as a stand-alone application that is cross-platform and database-independent.

## Discussion

We have built an application that facilitates annotation of minimotifs from the primary literature, which we are currently using to populate a more comprehensive MnM minimotif database. The application scores a set of papers for minimotif content. In principle, the TextMine score can be used to score all PubMed abstracts for minimotif content and can be used in the future for maintaining the database. As text mining algorithms increase in proficiency and scope, it may be possible to use a large, MimoSA-curated set of minimotif-containing papers as a training set for automatically detecting minimotif definition sentences and phrases in papers by machine learning approaches.

The implementation of the paper scoring algorithm as a SQL stored procedure in MimoSA automates its execution and is amenable to further machine learning development. A static algorithm would have required a word or file list as input and require manual merging of results into the database. One limitation of the TextMine application is that it does not directly control for type biasing. That is, depending on the training set, we expect that there is some risk of "weighting" words heavily to bias previously seen content types. Instead of controlling for this automatically, TextMine outputs the scores of all calculated words so as to enable user inspection of how their training set influences the algorithm. This allows for informed adjustments to the training set on a case-by-case basis.

Although MimoSA was developed primarily for Minimotif annotation, the PS algorithm for scoring content in papers has broader applications. In consideration of its potential use, we have implemented it as a separate program, TextMine. For other annotation purposes, correlation scores for individual words from a training set of articles already known to either contain, or not-contain, the desired information are calculated. This results in a rank order for several thousands of words. For each single article, the PS algorithm then calculates a Pearson's Correlation Coefficient between two large linear sets: the score of each word in the aforementioned dictionary, and the corresponding enrichment of that word in the article's title and abstract. Despite the broad range of semantic methodologies for communication of peptide minimotif information, we still observed significant differentiation of the paper rankings when applied to the minimotif content papers.

## Conclusions

The MimoSA application interfaces with a normalized model of minimotif function, facilitating non-redundant annotation of minimotifs. The MimoSA user interface combines a set of features that facilitate annotation; including the browsing, sorting, creation, and modification of minimotif annotation entries. Additionally, interactive paper selection, a database driven Minimotif Annotation Form and literature browser, minimotif attribute based markup and highlighting of abstracts, the display of minimotif positions in protein sequences, and minimotif publication scoring and status tracking. MimoSA also features an adaptive, database-driven paper-ranking strategy that can be used to rank papers for minimotif content, which, when combined with the paper tracking module, represents an adaptive approach to literature scoring and content rating. The layered architecture, generalizable data model of minimotif functionality, and database driven application components enable MimoSA to be readily adapted for other molecular annotation projects.

## Availability and Requirements

**Project name**: Minimotif System for annotation

**Project home page**: mimosa.bio-toolkit.com, textmine.bio-toolkit.com

**Operating system(s)**: Platform independent

**Programming language**: Java

**Other requirements**: MySQL 5.0 or higher, Java Virtual Machine 1.6 or higher,

**License**: Open Source

**Any restrictions to use**: This paper must be referenced in any publication that uses MimoSA or TextMine, or any application that is developed based on these core applications.

## Authors' contributions

MRS, JV, and RJN were involved in preparation and editing of the manuscript. TM, SR, VK and MRG were also involved in editing the manuscript. JV, TM, DS, and RJN designed and implemented the software application. SR, MRS, JV and VK were involved in identifying the strategies for paper identification. KK calculated MIE scores. JV designed and implemented the Paper Scoring algorithm and TextMine application. All authors read and approved the final manuscript.

## Supplementary Material

Additional File 1 **Additional material**. Approach for identifying papers with minimotif content, automated markup of abstracts, and pseudocode for paper scoring algorithm Click here for file
