# Supplementary material for: MimoSA: a system for minimotif annotation
Source: BMC Bioinformatics. 2010 Jun 16;11:328. doi: 10.1186/1471-2105-11-328 (PMC2905367; doi:10.1186/1471-2105-11-328)
Supplement: Additional File 1 — Additional material. Approach for identifying papers with minimotif content, automated markup of abstracts, and pseudocode for paper scoring algorithm [file 1471-2105-11-328-S1.DOC]

**Additional Material**

**Identification of papers with minimotif content**

In our initial attempts to collect papers from the literature that have minimotif content, we tested several queries. To evaluate each query, a Minimotif Identification Efficiency (MIE) score was calculated. To determine this score, a subset, consisting of 10-20 randomly-selected papers chosen from the results of the search, was selected. MIE is simply the percentage of those papers that have minimotifs. Using MIE and other criteria, a search query is either accepted or rejected. Accepted queries are used to add papers to a paper list in the Minimotif database (**Additional Fig. 1**).

In addition to Keyword and MeSH term queries of PubMed, we used several other strategies to identify papers containing minimotif information. These included: author/affiliation searches that identified papers by authors (with their institutional affiliations) of minimotif data-containing papers already in the MnM database, regular expression searches which identified papers with abstracts that contain strings of peptide sequences or consensus sequences using regular expressions, reverse citation searches which identified papers referenced by papers already in the MnM, forward citation searches which identified papers that referenced a paper in the MnM database, journal selection identified which journals have higher probabilities of publishing minimotif papers, and publication year which was used to restrict searches to more recent papers in PubMed. Combined, these strategies were used to build a list of ~130,000 papers that had a MIE score of ~30%.

**
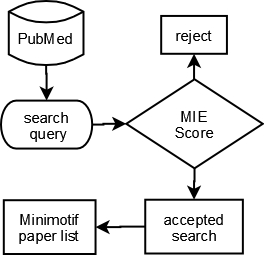
**

**Additional Figure 1**. **Strategy for identifying papers with minimotif content.**

**Automated markup of paper abstracts in MimoSA**

Through an integrated database of PubMed abstracts, their lexemes, and several million RefSeq and CDD keywords, MimoSA automates the process of marking up potential key annotation terms which are key indicators of minimotif meta data in abstracts [1, 2]. Automatically detectable elements of a minimotif annotation include activity terms, minimotif interaction domains, minimotif target or source proteins, and minimotif sequence information.

*Minimotif Detection.* In order to detect terms that might contain minimotif sequence or consensus residue information, we derived a regular expression for amino acid sequences. To speed up the process of minimotif sequence detection by users, papers are automatically screened, and all text sequences that conform to the following sequence by regular expression are highlighted and flagged.

((([Xx])|(Gly)|(Ala)|(Val)|(Leu)|(Ile)|(Met)|(Phe)|(Trp)|(Pro)|(Ser)|(Thr)|(Cys)|(Tyr)|(Asn)|(Gln)|(Lys)|(Arg)|(His)|(Asp)|(Glu)|(Lys)|(Thr)|(Trp)|\p{Punct}|)-?){3,15}.

*Activity Detection.* **To speed up the process of activity annotation,** key terms for suggested minimotif activities in a paper are automatically highlighted. These terms come from the several hundred discrete sub-activity term definitions in the MnM database. In addition, the words “binds”, “modifies”, and “required” are highlighted.

*Interaction Partners and Targets.* In order to detect important domains and / or proteins, a string-matching algorithm that searches for words which are associated with gene names, aliases, or RefSeq protein names is applied to all abstracts. Domains and proteins are highlighted in different colors. This was useful for annotation as many targets of minimotifs are proteins and more specifically domains within proteins.

**Pseudocode for paper scoring algorithm**

The pseudo code of our algorithm for our ranking methodology is show below.

-Given: T, a set of training articles represented as pairs of articles and positive indicator scores where a score of 0 indicates that the article contains no relevant data and a score of 1 indicates that the article contains relevant data For example:

(article 1, 0)

(article 2, 1)

(article 3, 1)

(article 4, 1)

(article 5, 0)

(article 6, 1)

Articles 2, 3, 4, and 6 all are highly relevant to the content being scored for, and articles 1 and 5 do not have relevant content.

-Given: A method for determining that two words are equivalent, or equivalently, a method for normalizing the text in an abstract (i.e., removing non alphanumeric characters and making case uniform) so that the overall amount of unique words is reduced.

*For example: In the sentence “Peptide motif-binding functions for binding of SH3/SH2 domain containing proteins.” Would normalize to “PEPTIDE MOTIF BINDING FUNCTIONS FOR BINDING OF SH3 SH2 DOMAIN CONTAINING PROTEINS”.*

The algorithm pseudo-code is as follows.

Generation of Word Scores from article summaries / training values.

Define t, u, and v as maps where the keys are strings and the values are integers. The sum of the scores will be stored in u, and the number of times each word has appeared will be stored in v.

For each article “a” and score "s" in T:

For each word “w” in a:

Increment u[w] by s

Increment v[w] by 1

Calculate the average score for each word:

For each word "w" in u:

t[w] = u[w] / v[w]

Define x: a map of articles to scores

Define y: a map where the keys are strings and the values are integers. This will be used to count the number of appearances of each word.

For each article "a" in the test set to be scored:

For each word "w" in "a":

Increment y[w] by 1

Using y and t, calculate the Pearson correlation coefficient for a

Set x[a] equal to (Pearson correlation coefficient for a / number of words in a)

Contained in x are the scores for the papers in the test set. Higher scores indicate a greater likelihood of relevance with respect to the content positively scored in the training set.

**Additional References**

1. Pruitt KD, Maglott DR: **RefSeq and LocusLink: NCBI gene-centered resources**. *Nucleic Acids Res* 2001, **29:**137-140.

2. Marchler-Bauer A, Anderson JB, DeWeese-Scott C, Fedorova ND, Geer LY, He S, Hurwitz DI, Jackson JD, Jacobs AR, Lanczycki CJ, Liebert CA, Liu C, Madej T, Marchler GH, Mazumder R, Nikolskaya AN, Panchenko AR, Rao BS, Shoemaker BA, Simonyan V, Song JS, Thiessen PA, Vasudevan S, Wang Y, Yamashita RA, Yin JJ, Bryant SH: **CDD: a curated Entrez database of conserved domain alignments**. *Nucleic Acids Res* 2003, **31:**383-387.
